# Effectiveness of *Tai Ji Quan* vs Multimodal and Stretching Exercise Interventions for Reducing Injurious Falls in Older Adults at High Risk of Falling: Follow-up Analysis of a Randomized Clinical Trial

**DOI:** 10.1001/jamanetworkopen.2018.8280

**Published:** 2019-02-15

**Authors:** Fuzhong Li, Peter Harmer, Elizabeth Eckstrom, Kathleen Fitzgerald, Li-Shan Chou, Yu Liu

**Affiliations:** 1 Oregon Research Institute, Eugene, Oregon; 2 Department of Exercise and Health Science, Willamette University, Salem, Oregon; 3 Division of General Internal Medicine and Geriatrics, School of Medicine, Oregon Health and Science University, Portland; 4 Oregon Medical Group Neurology, Eugene; 5 Department of Human Physiology, University of Oregon, Eugene; 6 School of Kinesiology, Shanghai University of Sport, Shanghai, China

## Abstract

**Importance:**

Exercise has been shown to reduce injurious falls in older adults. Evidence, however, is lacking regarding the types of intervention that are most effective in preventing injurious falls among older adults at high risk of falling.

**Objective:**

To determine the longer-term effectiveness of therapeutic *tai ji quan* intervention vs multimodal exercise and stretching exercise in decreasing injurious falls among older adults at high risk of falling.

**Design, Setting, and Participants:**

This trial involves a prespecified analysis with the data analyzed by intent-to-treat. Follow-up analysis of a single-blind randomized trial conducted in community settings of 7 urban and suburban cities in Oregon from February 20, 2015, to September 15, 2018, compared a therapeutic *tai ji quan* intervention with multimodal exercise and stretching exercise. Eligible participants were community-dwelling adults aged at least 70 years who were considered by a clinician to be at high risk of falling because they had fallen during the preceding year or who had impaired mobility with scores higher than 13.5 seconds on the Timed Up & Go test. Participants were randomized to 1 of the 3 interventions and were assessed monthly after randomization for 12 months, encompassing a 6-month active intervention phase and a 6-month after intervention follow-up phase.

**Interventions:**

The 3 group-based interventions were therapeutic *tai ji quan* (*Tai Ji Quan*: Moving for Better Balance [TJQMBB]), multimodal exercise, and stretching exercise, each implemented twice weekly in 60-minute sessions for 24 weeks.

**Main Outcomes and Measures:**

Primary outcomes were the incidence of moderate and serious injurious falls at 12 months, measured as incidence rate ratios (IRRs).

**Results:**

Of the 1147 persons screened, 670 (mean [SD] age, 77.7 [5.6] years; 436 women [65.1%]) were randomly assigned to 1 of 3 intervention groups: 224 persons in TJQMBB, 223 in multimodal exercise, and 223 in stretching exercise. At 12 months, the unadjusted IRR for moderate injurious falls was lower in the TJQMBB (IRR, 0.51; 95% CI, 0.35-0.74; *P* < .001) and multimodal exercise (IRR, 0.62; 95% CI, 0.42-0.89; *P* = .01) groups compared with the stretching exercise group. There was no difference between TJQMBB and multimodal exercise groups (IRR, 0.85; 95% CI, 0.58-1.25; *P* = .42). Both TJQMBB and multimodal exercise significantly reduced serious injurious falls (TJQMBB: IRR, 0.25 [95% CI, 0.13-0.48; *P* < .001]; multimodal: IRR, 0.56 [95% CI, 0.33-0.94; *P* = .03]) compared with stretching exercise. Use of TJQMBB was more effective than multimodal exercise (IRR, 0.47; 95% CI, 0.24-0.92; *P* = .03) in reducing serious injurious falls.

**Conclusions and Relevance:**

For preventing injurious falls, including those that resulted in medical treatment, TJQMBB intervention was found to be superior to multimodal and stretching exercises for older adults at high risk of falling. The findings appear to strengthen the clinical use of TJQMBB as a single exercise intervention to prevent injurious falls in this population.

**Trial Registration:**

ClinicalTrials.gov Identifier: NCT02287740

## Key Points

**Question:**

Is a therapeutically tailored *tai ji quan* intervention more effective than stretching exercise or a proven multimodal exercise intervention in decreasing injurious falls among community-dwelling older adults at high risk of falling?

**Findings:**

In this follow-up analysis of a randomized clinical trial that included 670 older adults with a history of falls or impaired mobility, a therapeutic *tai ji quan* intervention, *Tai Ji Quan*: Moving for Better Balance, was significantly more effective for lowering the incidence of both moderate injurious falls compared with stretching exercise and serious injurious falls compared with stretching exercise and multimodal exercise.

**Meaning:**

The therapeutically tailored *tai ji quan* training intervention proved to be more safe and effective than stretching or multimodal exercises in reducing the incidence of injurious falls in older adults at high risk of falling.

## Teaser

This follow-up analysis of a randomized clinical trial compares the effectiveness of *tai ji quan*, multimodal exercises, and stretching exercise interventions for older adults at high risk of falling.

## Introduction

Falls among older adults are a major cause of injury morbidity that can require medical treatment or lead to death.^1^ The significance of the problem is exemplified by the 30% increase in the mortality rate between 2007 and 2016 for older adults who fell.^2^ Treatment for fall-related injuries among older adults is costly.^3,4,5^ In 2015, the total medical costs for falls in this population in the United States was more than $50 billion.^5^ Therefore, preventing injurious falls has become a public health priority and is of critical importance for health care systems contending with the financial burden associated with fall-related injuries among older adults.

Many falls resulting in medical treatment can be prevented and the associated costs averted through community-based balance-training interventions.^6^ Increasing evidence suggests that exercise interventions reduce the incidence of falls and injurious falls among older adults^7,8,9,10^ and are, therefore, recommended for older adults with a high risk of falling.^11,12^ However, it is less clear which interventions are most effective in reducing injurious falls without exacerbating the risk in some individuals.^9^ Evidence is also scarce as to whether interventions with intermediate effects can reduce the long-term (postintervention) risk of injurious falls.^9^ All of these issues can impede the prescribing of exercise-based fall prevention interventions in clinical practice.

A recent 6-month randomized clinical trial^13^ (trial protocol is given in Supplement 1) showed that both a therapeutically based *tai ji quan* program and a multimodal exercise intervention significantly reduced the incidence of falls among older adults at high risk of falling. The study, however, did not examine whether the incidence of injurious falls was affected. Using data from the 6-month intervention and a 6-month follow-up of this clinical trial,^13^ we aimed to determine the longer-term postintervention effect of 2 exercise interventions, *Tai Ji Quan*: Moving for Better Balance (TJQMBB) and a multimodal exercise program compared with a stretching exercise intervention with the incidence of injurious falls in older adults at high risk of falling. Our predefined hypotheses were that, at follow-up, we would find sustained effects of TJQMBB and multimodal exercise with reductions in injurious falls, with a stronger effect anticipated from TJQMBB.

## Methods

### Study Design

This study was a follow-up analysis of a single-blind, 6-month randomized clinical trial in which participants were randomly allocated to 1 of 3 active arms: TJQMBB, multimodal exercise, or a stretching exercise control. After completing the 6-month active intervention, participants were followed up for 6 months after intervention, during which no structured activities were prescribed. Details of the trial design, eligibility requirements, randomization procedures, and exercise training protocols have been described elsewhere.^13^ Written informed consent was received from all participants, and the trial was approved by the institutional review board of the Oregon Research Institute, Eugene, Oregon, and overseen by a Data and Safety Monitoring Board appointed by the National Institute of Aging. Study recruitment lasted from February 20, 2015, to August 29, 2017, with the final study follow-up completed on September 15, 2018. The current study was conducted in community settings across 7 urban and suburban cities in Oregon and followed the Consolidated Standards of Reporting Trials (CONSORT) reporting guideline.

### Participants

As described previously,^13^ participants were recruited through posted flyers at local community centers, newspaper advertisements, medical clinics, and targeted mass mailings. Persons were eligible for the study if they were 70 years or older and met 1 of the 2 key eligibility criteria: (1) having fallen at least once in the preceding 12 months and having a clinician’s referral indicating the participant was at risk of falling or (2) having impaired mobility (Timed Up & Go^14^ >13.5 seconds^15^). See eAppendix 1 in Supplement 2 for additional criteria.

### Randomization and Masking

Participants were assigned randomly to receive 1 of the 3 interventions (TJQMBB, multimodal exercise, or stretching exercise) in a 1:1:1 allocation via a computer-generated randomization sequence with random permuted blocks of 3 or 6 participants. The allocation sequence was concealed to investigators, interventionists, and participants. Randomization took place after the baseline evaluation in 19 consecutive enrollment waves. Study assessors were masked to group allocation and remained masked throughout the study.

### Interventions

*Tai Ji Quan*: Moving for Better Balance involved practice of a core of 8 modified *tai ji quan* exercise forms with built-in variations and a subroutine of integrated therapeutic movement exercises.^16^ The practice emphasized synchronized breathing and exercise forms performed with weight shifting, unilateral weight bearing, head-shoulder-trunk alignment and rotation, and coordinated eye-head-hand movements with integration of self-initiated (proactive) and self-induced (reactive) movement perturbations. The multimodal exercise intervention, modified from a previous study,^17^ involved a mix of aerobic conditioning and strength, balance, and flexibility activities. Strength exercises included the use of hand weights, resistance tubing, and ankle weights; balance exercises included balance foams. All equipment was added in the past 3 months of the multimodal intervention. Modifications were made on an individual basis in both TJQMBB and multimodal exercise to accommodate the specific needs and performance capabilities of the participants. The stretching exercise, serving as an exercise control, consisted primarily of breathing, stretching, and relaxation activities, with most performed in a seated position. The full protocol for each of the 3 interventions is described elsewhere.^13^

### Intervention Shared Elements

Participants in each intervention group participated for 24 weeks in twice weekly training sessions that were 60 minutes. In all 3 groups, each exercise session consisted of 10 to 15 minutes of warm-up exercises, 40 to 45 minutes of core exercises, and 5 minutes of cool-down activities. No additional in-home or between-session exercises were assigned for any of the 3 interventions during the active intervention period.

### Postintervention Follow-up

After completing the active intervention, participants were followed up for 6 months, during which they were given no specific instructions about exercise but were encouraged to continue to exercise in any form. Participant levels of weekly physical activity were monitored using a home-exercise log,^18^ in which participants were asked to record the type, frequency, and duration of exercise performed. The information was ascertained at 3 and 6 months after the intervention. Research staff maintained once per month contact with the participants during the follow-up period to collect data about falls. The number of contacts and contact times were kept consistent across the 3 study groups.

### Outcomes

The primary outcome measures were the number of moderate and serious injurious falls during the 12 months. Falls were recorded using a daily “fall calendar”^13,19^ in which participants were asked to report any *fall incident* (defined as “when you land on the floor or the ground or fall and hit objects like stairs or pieces of furniture by accident”). If a fall was reported, participants were also asked to indicate whether they sought medical attention.

In this study, moderate injurious falls were defined as those that resulted in sprains, strains, contusions, or abrasions requiring no medical care. Serious injurious falls were those that resulted in admission to an emergency department or hospital. The initial sources of any injurious falls ascertainment were the fall calendar completed by the study participants and monthly phone contacts by study assessors. Information about each type of medical visit (hospital or emergency department) was verified, when possible, by health-related utilization expenses and/or medical records obtained from participants collected at 4, 6, and 12 months. Secondary outcomes were the number of fall-related emergency department visits and hospitalizations. Data on injurious falls were collected starting from the date of the first intervention class and continuing until 12 months later (ie, the last day of the study enrollment) or until a participant withdrew, died, or was lost to follow-up.

Baseline demographic measures, including age, sex, educational level, race/ethnicity, number of falls in the 6 months before intervention, resting blood pressure, and health status were collected.

### Statistical Analysis

The original study^13^ was powered with a sample size of 666 (including a planned 15% attrition rate) to detect a 35% reduction in fall incidence rate across a 6-month active intervention period between either of the 2 interventions (TJQMBB or multimodal exercise) compared with stretching exercise. Although no a priori sample size calculations for this follow-up study were conducted, on the basis of preliminary estimates obtained from a previous trial,^19^ we conservatively estimated a 30% reduction in moderate injurious falls and a 20% reduction in serious injurious falls for TJQMBB and multimodal exercise compared with stretching exercise. No specific prediction on reducing injurious falls was made between TJQMBB and multimodal exercise.

Descriptive analyses were performed among participant characteristics at baseline across interventions. Comparisons of group characteristics at baseline were undertaken using a χ^2^ test for differences in proportions and analysis of variance for differences in means. Analysis of variance was conducted to examine between-group differences in mean change from baseline to 12 months in primary and secondary outcomes. We also determined the rate of both moderate and serious injurious falls during 12 months, calculated using the number of falls observed from each participant as the numerator and their follow-up time as the denominator. In our primary analyses comparing the number of injurious falls with both primary and secondary outcomes, we used a priori specified negative binomial regression models to estimate absolute differences in incidence rate ratios (IRRs) from baseline to 12 months with their corresponding 95% CIs to compare TJQMBB and multimodal exercise with stretching exercise and TJQMBB and multimodal exercise. As part of planned group comparisons, we conducted additional analyses to test whether there was a difference between the injurious fall rates at the end of the 6-month active intervention and at the 6-month postintervention follow-up between the groups.

The IRRs were adjusted for important covariates (age, sex, number of falls 6 months before the start of intervention, health status, and levels of weekly physical activity at home ascertained at 12 months) and without these covariates. All analyses were based on intent-to-treat and adjusted for the variable follow-up times of participants. The significance level for the primary and secondary outcomes was *P* < .05 (2-tailed), without adjustment for multiple comparisons. Analyses were conducted using SPSS, version 23 (IBM Corp).

## Results

### Descriptive Statistics

Of the 1147 persons screened, 670 were randomly assigned to 1 of the 3 intervention groups: 224 adults in the TJQMBB group, 223 in the multimodal exercise group, and 223 in the stretching exercise group (eFigure in Supplement 2). The mean (SD) age of participants was 77.7 (5.6) years, and 436 (65.1%) were women. Baseline characteristics of the study participants were similar in the 3 randomized exercise interventions (Table 1).^13^

**Table 1.** Participant Characteristics at Baseline

Characteristic	TJQMBB Group (n = 224)	Multimodal Exercise Group (n = 223)	Stretching Exercise Group (n = 223)	*P* Value
Age, mean (SD), y	77.5 (5.6)	77.8 (5.3)	77.8 (5.9)	.83
Female sex, No. (%)	146 (65.2)	143 (64.1)	147 (65.9)	.92
Race/ethnicity, No (%)				
Hispanic	24 (10.7)	24 (10.8)	23 (10.3)	.99
Non-Hispanic	200 (89.3)	199 (89.2)	200 (89.7)	
American Indian or Alaska Native	1 (0.4)	0	2 (0.9)	.34
Asian	2 (0.9)	3 (1.3)	1 (0.4)	
Black or African American	13 (5.8)	14 (6.3)	4 (1.8)	
Native Hawaiian or other Pacific Islander	1 (0.4)	0	2 (0.9)	
White	203 (90.6)	203 (91.0)	211 (94.6)	
Multiracial/no response	4 (1.8)	3 (1.3)	3 (1.3)	
Educational level, No. (%)				
High school diploma or lower	95 (42.4)	101 (45.3)	92 (41.3)	.67
College degree or higher	129 (57.6)	122 (54.7)	131 (58.7)	
Resting blood pressure, mean (SD), mm Hg				
Systolic	133.3 (19.3)	135 (19.7)	134.1 (19.1)	.56
Diastolic	75.6 (11.3)	75.6 (10.5)	74.2 (11.5)	.33
Body mass index, mean (SD)^a^	29.2 (6.0)	29.4 (6.6)	29.91 (6.2)	.48
Self-reported falls in previous 6 mo, No. (%)				
None	63 (28.1)	61 (27.4)	61 (27.4)	.80
1	79 (35.3)	71 (31.8)	72 (32.3)	
≥2	82 (36.6)	91 (40.8)	90 (40.3)	

Abbreviation: TJQMBB, *Tai Ji Quan*: Moving for Better Balance.

^a^ Calculated as weight in kilograms divided by height in meters squared.

The 6-month intervention had a 13.0% attrition rate (defined as persons who dropped out of the intervention voluntarily but continued to provide outcome data). By month 12 (6 months after intervention follow-up), 36 participants (5.4%) had discontinued participation in the study (defined as persons who dropped out of the intervention voluntarily and declined to provide any follow-up data). Data on the primary outcomes of moderate and serious injurious falls were available for 669 participants (99.9%) at 6 months (termination of intervention) and 634 participants (94.6%) at 12 months (postintervention follow-up). The mean (SD) follow-up time for the study population was 11.71 (1.24) months, with a total of 7849 person-months (2649 person-months for TJQMBB, 2619 person-months for multimodal exercise, and 2581 person-months for stretching exercise).

### Intervention Adherence

The overall class attendance rate (the sum of the total number of participants attending divided by the maximum number of 48 sessions planned, multiplied by 100) during the 6 months of active intervention was 77.3%. The mean rate of attendance across the 3 intervention groups was similar.^13^ A total of 158 participants in the TJQMBB group (70.5%), 160 in the multimodal exercise group (71.7%), and 159 in the stretching exercise control group (71.3%) attended 37 or more sessions (>75.0% of the planned total intervention class sessions).

### Serious Adverse Events

Adverse events were closely monitored and ascertained at each contact with the patient and verified through medical records, if available, during the study period (eAppendix 2 in Supplement 2). There were no serious adverse events related to the interventions.

### Exercise Behaviors at the Postintervention Follow-up

Continuing exercise data during the 6-month postintervention period across the 3 intervention groups are presented in eTable 1 in Supplement 2. At the postintervention follow-up, 431 participants (64.3%) reported engaging in exercise at least 30 minutes weekly (143 participants in the TJQMBB group, 141 participants in the multimodal exercise group, and 147 participants in the stretching exercise group), with a mean (SD) of 135.23 (225.04) minutes per week. There were no between-group differences in the total amount of exercise behavior observed during the postintervention follow-up (*F*_2,667_ = 0.29; *P* = .75).

### Primary Outcomes

Table 2 presents descriptive statistics of the data on injurious falls ascertained during the 12-month study. Of the total of 102 serious injurious falls documented, 90 (88.2%) (11 in the TJQMBB group, 29 in the multimodal exercise group, and 50 in the stretching exercise group) were verified using objective medical records (ie, medical insurance or Medicare billing statements) collected from the participants. There was a significant between-group difference in the mean number of moderate injurious falls (*F*_2,667_ = 3.9; *P* = .02). Post hoc analyses showed that the TJQMBB group had a significantly lower mean (SD) number of moderate injurious falls than the stretching exercise group (0.57 [1.28] vs 1.04 [2.42]; *P* = .04). There was also a between-group difference in serious injurious falls (*F*_2,667_ = 9.26; *P* = .001), with the TJQMBB group (0.07 [0.27]) and the multimodal exercise group (0.14 [0.44]) having a significantly lower mean (SD) number of falls compared with the stretching exercise group (0.25 [0.56]) (*P* = .04).

**Table 2.** Descriptive Statistics for the Number of Injurious Falls at 6 and 12 Months by Intervention Group

Descriptive Statistic	TJQMBB Group (n = 224)	Multimodal Exercise Group (n = 223)	Stretching Exercise Group (n = 223)	*P* Value
**6 mo**				
Injurious falls, No.				
Moderate^a^	88	109	156	.04
Serious^b^	8	14	25	.02
**12 mo**				
Injurious falls, No.				
Moderate^a^	127	148	232	.02
Serious^b^	15	32	55	.001
Person-months of observation	2649	2619	2581	NA
Incident rate for moderate injurious falls, person-months	47.9	56.5	89.9	NA
Incident rate for serious injurious falls, person-months	5.6	12.2	21.3	NA

Abbreviations: NA, not applicable; TJQMBB, *Tai Ji Quan*: Moving for Better Balance.

^a^ Defined as falls that resulted in sprains, strains, contusions, or abrasions that required no professional medical care. None of these injuries was related to the intervention.

^b^ Defined as falls that resulted in admission to an emergency department or hospital. None of these injuries was related to the intervention.

Results of analyses of the association between the number of injurious falls and intervention group during the 12 months are presented in Table 3. At 12 months, compared with the stretching exercise group, unadjusted IRR for moderate injurious falls was lower in the TJQMBB group (IRR, 0.51; 95% CI, 0.35-0.74; *P* < .001) and multimodal exercise group (IRR, 0.62; 95% CI, 0.42-0.89; *P* = .01). There was no statistically significant difference between the TJQMBB group and the multimodal exercise group (IRR, 0.85; 95% CI, 0.58-1.25; *P* = .42). The TJQMBB and multimodal exercise group had a lower incidence rate of serious injurious falls compared with the stretching exercise group (TJMBB group: IRR, 0.25 [95% CI, 0.13-0.48; *P* < .001]; multimodal exercise group: 0.56 [95% CI, 0.33-0.94; *P* = .03]); TJQMBB was significantly more effective in reducing serious injurious falls than multimodal exercise (IRR, 0.47; 95% CI, 0.24-0.92; *P* = .03). The results on serious injurious falls were consistent across comparisons when 12 participants with no objective medical data were removed (4 in the TJQMBB group, 3 in the multimodal exercise group, and 5 in the stretching exercise group).

**Table 3.** Number of Injurious Falls and Intervention Group During 12 Months Using Stretching Exercise as a Reference Group^a^

Outcome	Unadjusted IRR (95% CI)	*P* Value	Adjusted IRR (95% CI)^b^	*P* Value
**Primary**				
Moderate injurious falls				
TJQMBB vs stretching exercise	0.51 (0.35-0.74)	<.001	0.53 (0.36-0.76)	.001
Multimodal exercise vs stretching exercise	0.62 (0.42-0.89)	.01	0.65 (0.45-0.94)	.02
Serious injurious falls				
TJQMBB vs stretching exercise	0.25 (0.13-0.48)	<.001	0.25 (0.13-0.46)	<.001
Multimodal vs stretching exercise	0.56 (0.33-0.94)	.03	0.53 (0.32-0.88)	.02
**Secondary**				
Emergency department visits				
TJQMBB vs stretching exercise	0.26 (0.12-0.52)	<.001	0.26 (0.14-0.51)	<.001
Multimodal vs stretching exercise	0.55 (0.31-0.97)	.04	0.52 (0.30-0.91)	.02
Hospitalizations				
TJQMBB vs stretching exercise	0.27 (0.10-0.73)	.01	0.26 (0.10-1.71)	.008
Multimodal vs stretching exercise	0.60 (0.28-1.29)	.19	0.58 (0.27-1.24)	.16

Abbreviations: IRR, incidence rate ratio; TJQMBB, *Tai Ji Quan*: Moving for Better Balance.

^a^ Intervention groups: TJQMBB (n = 224), stretching exercise (n = 223), and multimodal exercise (n = 223).

^b^ Analyses included variables of age, sex, number of baseline falls, health status assessed at baseline, levels of weekly physical activity after intervention follow-up assessed at 12 months, and participant follow-up time.

### Secondary Outcomes

#### Emergency Department Visits

A total of 68 injury-related visits to the emergency department (reported by 61 participants) were documented throughout the 12 months of the study (10 [3.8 per 1000 person-months] in the TJQMBB, 21 [8 per 1000 person-months] in the multimodal exercise group, and 37 [14 per 1000 person-months] in the stretching exercise group; *P* < .001). The unadjusted IRR for emergency department visits was lower in the TJQMBB group (0.26; 95% CI, 0.12-0.52; *P* < .001) and multimodal exercise group (0.55; 95% CI, 0.31-0.97; *P* = .04) compared with the stretching exercise group (Table 3). There was, however, no statistically significant difference between TJQMBB and multimodal exercise (IRR, 0.47; 95% CI, 0.21-1.07, *P* = .07) with emergency department visits.

#### Hospitalizations

A total of 34 fall-related hospital admissions were reported by 32 participants throughout the 12 months of the study (5 [1.9 per 1000 person-months] in the TJQMBB group, 11 [4 per 1000 person-months] in the multimodal exercise group, and 18 [7 per 1000 person-months] in the stretching exercise group; *P* = .03). Negative binominal regression analyses showed that TJQMBB had a lower hospital admission rate relative to stretching (IRR, 0.27; 95% CI, 0.10-0.73; *P* = .01), and there was no significant reduction in hospital admission rate for multimodal exercise compared with stretching exercise (IRR, 0.60; 95% CI, 0.28-1.29; *P* = .19) (Table 3). There was also no statistically significant difference between the associations of TJQMBB and multimodal exercise with hospital admission rate (IRR, 0.47; 95% CI, 0.21-1.07; *P* = .14).

### Additional Analyses

Estimates from the negative binomial regression models analyzing between-group differences during the 6-month active intervention and 6-month postintervention follow-up phases are presented in eTable 2 in Supplement 2. Results suggest that TJQMBB was effective compared with stretching exercise in reducing both moderate and serious injurious falls in both phases of the study. There was also a significant reduction in the incidence of serious injurious falls during the 6-month postintervention follow-up in the TJQMBB group compared with the multimodal exercise group (IRR, 0.33; 95% CI, 0.14-0.81; *P* = .02). Compared with the stretching exercise, multimodal exercise was effective in reduced incidence of moderate and serious injurious falls during the 6-month active intervention phase and only with reduced incidence of moderate injurious falls during the 6-month postintervention follow-up. An unplanned analysis on the biologic variable of sex showed no differences between women and men in responding to primary and secondary intervention outcomes.

## Discussion

Previous study findings^13^ showed that, compared with stretching exercise, a 6-month TJQMBB intervention and a multimodal exercise intervention significantly reduced the incidence of falls among community-dwelling older adults who were at high risk of falls. In this 12-month follow-up study, the results showed that, although both exercise interventions were associated with a reduced incidence of moderate and serious injurious falls compared with stretching exercise, TJQMBB was shown to be more effective (by 53.0%) compared with the multimodal exercise intervention. The effect of TJQMBB with reduced incidence of falls was consistent and robust, as evidenced by the sustained reduction observed during the 6-month postintervention follow-up.

Results from this study were congruent with findings from recent systematic reviews and meta-analyses about fall prevention.^7,8,9,10^ These review studies showed that, among various common interventions, exercise compared with usual care or controls was associated with reductions in falls and injury-related falls in older adults who were at increased risk for falls. However, lack of a tailored exercise approach has produced conflicting findings that have made it difficult to recommend the type of exercise best suited for reducing injurious falls.^9^ To address this problem, this study provided information regarding the comparative effectiveness of 2 different but increasingly common types of exercise interventions (equipment nondependent vs equipment dependent or balance-tailored vs general exercises) for reducing injurious falls among older adults who were at high risk of falling.

Few fall prevention interventions are specifically tailored toward enhancing balance and reducing injurious falls.^9^ As one of the few interventions, TJQMBB targets modifiable fall risk factors (eg, impaired balance, reduced lower-extremity strength, diminished limits of stability, and gait abnormalities). From an implementation perspective, as a group-based instructor-led program, TJQMBB uses a manual-driven, progressive approach that walks participants through fundamental motor skills related to the performance of various *tai ji quan* forms and mini-therapeutic exercises.^16^ The program is readily implementable and scalable and easily disseminated in community settings^20,21,22,23,24^ and clinical practice through patient referrals to community-based classes,^25^ features that facilitate program outreach and uptake among clinicians who provide primary care to populations who are at high risk of falling.^26,27^

Findings from this study have implications for primary care and geriatric clinicians and individuals planning programs for improving community care of older adults at high risk of falling by reducing their fall-related injuries. First, all study participants were medically cleared through their clinicians, which indicates that patients who are at high risk of falling can be recruited from health care delivery systems into community-based fall prevention programs.^26^ Second, instead of exercising at a standard recommendation of 3 hours each week,^8^ findings from this study suggest that referring community-dwelling older adults who have a history of falling or who have clinically identifiable balance issues or impaired mobility (ie, Timed Up & Go >13.5 seconds) for participation in this non-equipment-based, therapeutically tailored, 2 hours per week *tai ji quan* exercise program may have considerable influence for preventing or reducing injurious falls. Finally, our study also suggests that although both TJQMBB and multimodal exercise are associated with reduced incidence of injurious falls, the former has the additional advantage of requiring few resources for program implementation (eg, no equipment required, low costs,^20^ and minimal space needed), thereby enhancing return on investment^28^ and cost-effectiveness.^29^

### Limitations

The study has limitations. First, moderate injurious falls were self-reported, and although we were able to identify serious injurious falls using medical records, not all serious falls were objectively verified. Therefore, some self-report bias likely exists in our data. Second, the 6-month postintervention follow-up data may not be indicative of the longer-term impact of TJQMBB or multimodal exercise with preventing injurious falls. Because our intervention was conducted with people living in the general community, our findings may not generalize beyond community-dwelling older adults to at-risk individuals in residential care settings or those having specific clinical conditions.^7,8^ Also, our primary analysis on reducing injurious falls was not powered a priori, which may reduce the sensitivity of the results. These findings should be confirmed in future studies.

## Conclusions

Among community-dwelling older adults at high risk of falls, during a 1-year period, TJQMBB compared with multimodal exercise and stretching exercise was associated with a reduced incidence of injurious falls, including those that resulted in adults seeking medical care. The findings strengthen the clinical utility of TJQMBB as a single exercise intervention to prevent injurious falls in this population.

## Notes

Supplement 1. **Trial Protocol**

Supplement 2. **eAppendix 1.** Additional Study Eligibility Criteria **eFigure.** Flow of Participant Recruitment, Randomization, and Follow-Up **eAppendix 2.** Serious Adverse Events **eTable 1.** Mean Weekly Minutes of Exercise Behaviors Reported by Participants During the 6-month Postintervention Period by Intervention **eTable 2.** Analyses by Study Phase (6-Month Active Intervention, 6-Month Post-Intervention Follow-Up)

Supplement 3. **Data Sharing Statement**
