# Data Error in Results

**DOI:** 10.1001/jamanetworkopen.2019.2314

**Published:** 2019-03-15

In the Original Investigation titled “Effectiveness of *Tai Ji Quan* vs Multimodal and Stretching Exercise Interventions for Reducing Injurious Falls in Older Adults at High Risk of Falling: Follow-up Analysis of a Randomized Clinical Trial,”^1^ published February 15, 2019, a data error occurred in the Intervention Adherence subsection of the Results section. The third sentence should have read, “A total of 158 participants in the TJQMBB group (70.5%), 160 in the multimodal exercise group (71.7%), and 159 in the stretching exercise control group (71.3%) attended 37 or more sessions (>75.0% of the planned total intervention class sessions).” This article has been corrected.^1^
