# Supplementary material for: Effectiveness of Tai Ji Quan vs Multimodal and Stretching Exercise Interventions for Reducing Injurious Falls in Older Adults at High Risk of Falling: Follow-up Analysis of a Randomized Clinical Trial
Source: JAMA Netw Open. 2019 Feb 15;2(2):e188280. doi: 10.1001/jamanetworkopen.2018.8280 (PMC6484587; doi:10.1001/jamanetworkopen.2018.8280)
Supplement: Supplement 1. — Trial Protocol [file jamanetwopen-2-e188280-s001.pdf]

Trial Protocol

Design

The study involved a single-blind 6-month randomized clinical trial in which participants were randomly allocated to one of three active intervention arms: *Tai Ji Quan: Moving for Better Balance* (TJQMBB), multimodal exercise, or a stretching exercise control. Details of the trial design, eligibility requirements, randomization procedures, and exercise training protocols during the 6-month active intervention period have been described in the study by Li et al. referenced below:

Li F, Harmer P, Fitzgerald K, Eckstrom E, Akers, L, Chou L-S, Pidgeon D, Voit J, Winters-Stone K. Comparative effectiveness of a therapeutic Tai Ji Quan intervention versus a multimodal exercise intervention to prevent falls among older adults at high risk of falling: a randomized clinical trial. *JAMA Intern Med* 2018;178(10):1310-1308.

Post-Intervention Follow-up.

As part of the original research protocol, upon completing the active intervention, participants were followed up for a duration of 6 months. During the follow-up, participants were given no specific instructions on exercise but they were encouraged to continue to exercise in any form. Participants' levels of weekly physical activity or exercise behaviors were monitored via a home-exercise log in which they were asked to record information on type, frequency, and duration of the exercise undertaken. Research staff maintained once per month contact with the participants during the follow-up period to collect data on falls. The number of contacts and contact times were kept consistent across the three intervention groups. The post-intervention follow-up protocol was approved by the Institutional Review Board of Oregon Research Institute, located in Eugene, Oregon.

Data Ascertainment

During the 6-month post-intervention follow-up, the following outcome measures were collected by means of fall calendars, monthly phone calls by research staff, and medical records:

1. Falls
2. Injurious falls including serious injurious falls (primary) and moderate injurious falls (secondary)
3. Other information included the number of emergency room visits and hospital admissions.

In addition, information on physical activity (exercise behaviors) was collected at 3-month intervals by research assistants who were blinded to intervention group allocation.

Data analysis

The original study was powered, with a sample size of 666 (including a planned 15% attrition rate), to detect a 35% reduction in fall incidence rate across a 6-month active intervention period (a respective IRR of 0.65) between either of the two interventions (TJQMBB, multimodal exercise) relative to stretching exercise. No a priori sample size calculations for the 6-month post-intervention follow-up study were conducted. However, on the basis of preliminary estimates obtained from a prior trial, conservative estimates of a 30% reduction in moderate injurious falls and a 20% reduction in serious injurious falls for TJQMBB and multimodal exercise relative to stretching exercise were established. No specific predictions on

reductions in injurious falls between TJQMBB and multimodal exercise interventions were made.

Analysis of variance was planned to examine between-group differences in mean change from baseline to 12 months in primary and secondary outcomes. We also present the rate of falls over 12 months, calculated using the number of falls observed from each participant as the numerator and their follow-up time as the denominator. In our primary analyses comparing the number of injurious falls, we used a priori specified negative binomial regression models to estimate absolute differences in incidence rate ratios (IRRs) from baseline to 12 months with their corresponding 95% confidence intervals (CIs) comparing TJQMBB and multimodal exercise with stretching exercise and TJQMBB and multimodal exercise. As part of planned group comparisons, we also conducted additional analyses to test whether there was a difference between the injurious fall rates at the end of the 6-month active intervention and at the 6-month post-intervention follow-up between the groups.

IRRs were adjusted for important covariates (age, sex, number of falls 6 months prior to the start of intervention, health status, and levels of weekly physical activity at home ascertained at 12 months) and without these covariates. All analyses were based on intent-to-treat (i.e., all participants who were enrolled and randomly allocated to interventions were analyzed in the groups to which they were randomized) and adjusted for the variable follow-up times of participants. Significance testing was 2-sided. Results were considered statistically significant if $P < 0.05$. No adjustment was made on multiple comparisons. Analyses were conducted using SPSS version 23 (IBM Corp, Armonk, NY).
