# Supplementary material for: Effectiveness of Tai Ji Quan vs Multimodal and Stretching Exercise Interventions for Reducing Injurious Falls in Older Adults at High Risk of Falling: Follow-up Analysis of a Randomized Clinical Trial
Source: JAMA Netw Open. 2019 Feb 15;2(2):e188280. doi: 10.1001/jamanetworkopen.2018.8280 (PMC6484587; doi:10.1001/jamanetworkopen.2018.8280)
Supplement: Supplement 2. — eAppendix 1. Additional Study Eligibility Criteria eFigure. Flow of Participant Recruitment, Randomization, and Follow-Up eAppendix 2. Serious Adverse Events eTable 1. Mean Weekly Minutes of Exercise Behaviors Reported by Participants During the 6-month Postintervention Period by Intervention eTable 2. Analyses by Study Phase (6-Month Active Intervention, 6-Month Post-Intervention Follow-Up) [file jamanetwopen-2-e188280-s002.pdf]

Supplementary Online Content

Li F, Harmer P, Eckstrom E, Fitzgerald K, Chou L-S, Liu Y. Effectiveness of *tai ji quan* vs multimodal and stretching exercise interventions for reducing injurious falls in older adults at high risk of falling: follow-up analysis of a randomized clinical trial. *JAMA Netw Open.* 2019;2(2):e188280. doi: 10.1001/jamanetworkopen.2018.8280

eAppendix 1. Additional Study Eligibility Criteria

eFigure. Flow of Participant Recruitment, Randomization, and Follow-Up

eAppendix 2. Serious Adverse Events

eTable 1. Mean Weekly Minutes of Exercise Behaviors Reported by Participants During the 6-month Postintervention Period by Intervention

eTable 2. Analyses by Study Phase (6-Month Active Intervention, 6-Month Post-Intervention Follow-Up)

This supplementary material has been provided by the authors to give readers additional information about their work.

© 2019 Li F et al. *JAMA Network Open.*

eAppendix 1. Additional Study Eligibility Criteria

As described in Li et al. (*JAMA Intern Med* 2018, doi:10.1001/jamainternmed.2018.3915), additional eligibility criteria for the study included the following:

Inclusion criteria:
(1) being able to walk 1 or 2 blocks, with or without the use of an assistive device,
(2) being able to exercise safely as determined by a healthcare provider, and
(3) having a willingness to be randomly assigned to an intervention condition and complete the 6-month intervention.

Exclusion criteria:
(1) participating in daily and/or structured vigorous physical activity or walking for exercise that lasted 15 minutes or longer or muscle-strengthening activities (e.g., weight lifting) on 2 or more days a week in the previous 3 months,
(2) having severe cognitive impairment (Mini-Mental State Examination [MMSE][1] score ≤20 on a range of 0 to 30), and
(3) major medical or physical conditions determined by their healthcare provider to preclude exercise.

Reference:
1. Folstein MF, Folstein SE, McHugh PR. Mini-Mental State: A practical method for grading the cognitive state of patients for the clinician. *J Psychiatrics Res*, 1975, 12(3):189-98.

© 2019 Li F et al. *JAMA Network Open.*

eFigure. Flow of Participant Recruitment, Randomization, and Follow-Up

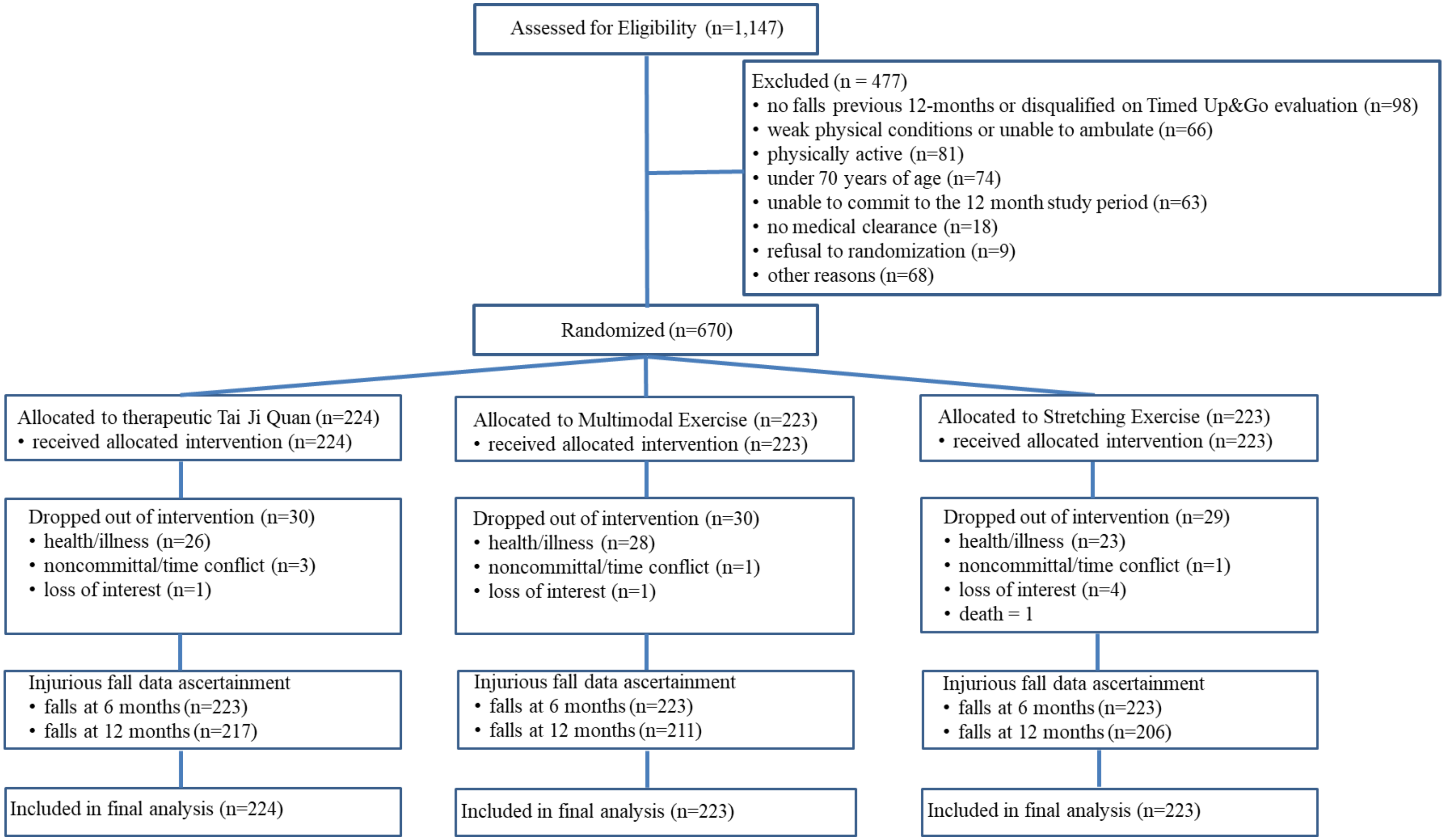

© 2019 Li F et al. *JAMA Network Open.*

eAppendix 2. Serious Adverse Events

In this study, we defined serious adverse events (SAE) as death or medical conditions that required one or more days of hospitalization. The data below identifies all SAE observed during the 12-months study. All SAE were reported to the Institutional Review Board that authorized the study and the Data and Safety Monitoring Board appointed by the National Institute on Aging. Following review by research staff, none of these SAE were determined to be related to the intervention. Other non-serious events observed during class sessions are also presented.

TJQMBB

SAEs: Seventeen participants reported hospital admissions. One death was documented during the 6-month post-intervention follow-up.

Ten participants were documented as having emergency department visits.

Two non-injurious falls from two independent participants were documented during a class session.

Multimodal exercise

SAEs. Sixteen participants reported hospital admissions. One death was documented during the 6-month post-intervention follow-up.

Twenty-one participants were documented as having emergency department visits.

Three non-injurious falls from three independent participants were documented during a class session.

Stretching exercise

SAEs. Nineteen participants reported hospital admissions. One death was documented during the 6-month intervention period and three deaths were documented during the 6-month post-intervention follow-up.

Thirty-eight participants were documented as having emergency department visits.

Two non-injurious falls from two independent participants were documented during a class session.

© 2019 Li F et al. *JAMA Network Open.*

eTable 1. Mean Weekly Minutes of Exercise Behaviors Reported by Participants During the 6-month Postintervention Period by Intervention

	TJQMBB	Multimodal exercise	Stretching exercise
Exercise, min/wk (SD)	141.55 ± 210.31	137.98 ± 244.88	126.12 ± 218.54

Abbreviation: TJQMBB, *Tai Ji Quan: Moving for Better Balance*.
Note: There were no between-group differences in the total amount of exercise behaviors observed during post-intervention follow-up, $F(2,667) = 0.29$, $p = 0.75$.

© 2019 Li F et al. *JAMA Network Open.*

eTable 2. Analyses by Study Phase (6-Month Active Intervention, 6-Month Post-Intervention Follow-Up)

The following table shows the results (both unadjusted and adjusted) of between-group differences (using stretching exercise as a reference) in injurious fall rates during the 6-month active intervention and 6-month postintervention follow-up.

Outcome		Incidence rate ratio (95% CI)		
	Unadjusted	P Value	Adjusted[a]	P Value
		**6-month active intervention**		
Moderate injurious falls				
TJQMBB vs stretching exercise	0.56 (0.37 to 0.83)	0.004	0.57 (0.39 to 0.85)	0.006
Multimodal vs stretching exercise	0.68 (0.45 to 0.99)	<0.05	0.72 (0.49 to 0.99)	<0.05
Serious injurious falls				
TJQMBB vs stretching exercise	0.32 (0.14 to 0.70)	0.005	0.31 (0.14 to 0.59)	0.006
Multimodal vs stretching exercise	0.43 (0.21 to 0.88)	0.03	0.43 (0.21 to 0.87)	0.02
	**6-month postintervention follow-up**			
Moderate injurious falls				
TJQMBB vs stretching exercise	0.49 (0.28 to 0.87)	0.02	0.48 (0.27 to 0.86)	0.01
Multimodal vs stretching exercise	0.50 (0.28 to 0.89)	0.02	0.49 (0.28 to 0.87)	0.02
Serious injurious falls				
TJQMBB vs stretching exercise	0.23 (0.10 to 0.55)	0.001	0.23 (0.10 to 0.54)	0.001
Multimodal vs stretching exercise	0.69 (0.37 to 1.28)	0.24	0.67 (0.37 to 1.23)	0.19

Abbreviation: TJQMBB, *Tai Ji Quan: Moving for Better Balance*.

[a]Negative bionominal regression model included covariates of age, sex, number of falls at baseline, health status assessed at baseline, and levels of weekly physical activity during postintervention follow-up (assessed at 12 months).

© 2019 Li F et al. *JAMA Network Open.*
