# Supplementary material for: Effectiveness of Tai Ji Quan vs Multimodal and Stretching Exercise Interventions for Reducing Injurious Falls in Older Adults at High Risk of Falling: Follow-up Analysis of a Randomized Clinical Trial
Source: JAMA Netw Open. 2019 Feb 15;2(2):e188280. doi: 10.1001/jamanetworkopen.2018.8280 (PMC6484587; doi:10.1001/jamanetworkopen.2018.8280)
Supplement: Supplement 3. — Data Sharing Statement [file jamanetwopen-2-e188280-s003.pdf]

Data Sharing Statement

Li. Effectiveness of Tai Ji Quan vs Multimodal and Stretching Exercise Interventions for Reducing Injurious Falls in Older Adults at High Risk of Falling. *JAMA Netw Open*. Published February 15, 2019. 10.1001/jamanetworkopen.2018.8280

Data
Data available: Yes
Data types: Deidentified participant data
How to access data: Fuzhong Li, Ph.D., at fuzhongl@ori.org
When available: With publication

Supporting Documents
Document types: Other (please specify)
Additional Information: the study protocol is described in a Supplement
How to access documents: as online supplement
When available: With publication

Additional Information
Who can access the data: researchers
Types of analyses: summary data
Mechanisms of data availability: with investigator support
